# Scaling Beyond Early Adopters: a Content Analysis of Literature and Key Informant Perspectives

**DOI:** 10.1007/s11606-020-06142-0

**Published:** 2020-10-27

**Authors:** Isomi Miake-Lye, Selene Mak, Christine A. Lam, Anne C. Lambert-Kerzner, Deborah Delevan, Tanya Olmos-Ochoa, Paul Shekelle

**Affiliations:** 1 VA Greater Los Angeles Healthcare System, Los Angeles, CA USA; 2 Fielding School of Public Health, University of California, Los Angeles, Los Angeles, CA USA; 3 David Geffen School of Medicine, University of California Los Angeles, Los Angeles, CA USA; 4 University of Colorado Denver, Denver, CO USA

**Keywords:** content analysis, interviews, scale-up, spread, late adopters, literature synthesis

## Abstract

**Background:**

Innovations and improvements in care delivery are often not spread across all settings that would benefit from their uptake. Scale-up and spread efforts are deliberate efforts to increase the impact of innovations successfully tested in pilot projects so as to benefit more people. The final stages of scale-up and spread initiatives must contend with reaching hard-to-engage sites.

**Objective:**

To describe the process of scale-up and spread initiatives, with a focus on hard-to-engage sites and strategies to approach them.

**Design:**

Qualitative content analysis of systematically identified literature and key informant interviews.

**Participants:**

Leads from large magnitude scale-up and spread projects.

**Approach:**

We conducted a systematic literature search on large magnitude scale-up and spread and interviews with eight project leads, who shared their perspectives on strategies to scale-up and spread clinical and administrative practices across healthcare systems, focusing on hard-to-engage sites. We synthesized these data using content analysis.

**Key Results:**

Searches identified 1919 titles, of which 52 articles were included. Thirty-four discussed general scale-up and spread strategies, 11 described hard-to-engage sites, and 7 discussed strategies for hard-to-engage sites. These included publications were combined with interview findings to describe a fourth phase of the national scale-up and spread process, common challenges for spreading to hard-to-engage sites, and potential benefits of working with hard-to-engage sites, as well as useful strategies for working with hard-to-engage sites.

**Conclusions:**

We identified scant published evidence that describes strategies for reaching hard-to-engage sites. The sparse data we identified aligned with key informant accounts. Future work could focus on better documentation of the later stages of spread efforts, including specific tailoring of approaches and strategies used with hard-to-engage sites. Spread efforts should include a “flexible, tailored approach” for this highly variable group, especially as implementation science is looking to expand its impact in routine care settings.

**Electronic supplementary material:**

The online version of this article (10.1007/s11606-020-06142-0) contains supplementary material, which is available to authorized users.

## INTRODUCTION

Moving research insights into clinical practice can be slow and a gap often remains between best practices, frequently developed within single sites or small populations, and care delivered at a population scale.^1–6^ The field of implementation science seeks to mend this gap by promoting the adoption and appropriate use of effective interventions, practices, policies, and programs in routine healthcare and public health settings.^7–10^ One growing facet within this large, interdisciplinary field is the study of scale-up and spread of innovations.^8, 11–13^ The terms “scale-up” and “spread” are not well-differentiated and often used together or interchangeably.^8, 14^ An exemplar definition describes scale-up and spread as “deliberate efforts to increase the impact of innovations successfully tested in pilot or experimental projects so as to benefit more people and to foster policy and program development on a lasting basis.”^8, 15^ This example exhibits typical components of scale or spread definitions, including the pre-established effectiveness of the innovation; the expansion across systems, sites, or settings; and the intentional process or active effort involved.^1, 8, 12, 14, 16^

Numerous frameworks and models have been developed for scale-up and spread,^1–4, 9, 14, 17–20^ with a recent review identifying 24 concepts, theories, or models in the public health sector alone.^16^ Here we focus on 2 widely used frameworks that describe the process of multisite scale-up and spread: the Institute for Healthcare Improvement’s phases of scale-up^1^ and the QUERI pipeline.^21^ These frameworks follow three similar steps in the spread process: piloting and initial testing of some idea or innovation, small-scale test of spread strategies scale-up, and full scale-up or spread. Whether the earliest stage includes using an evidence-based innovation^21^ or developing a new idea,^1^ the first phase includes small-scale testing or piloting with direct involvement of the team at the initial site or small number of sites. This work requires personalized, first-hand contact and typically builds relationships among those developing, implementing, and evaluating the initiative. After initial testing, the regional roll-out phase allows for small-scale test of implementation strategies^22^ for scale-up or spread strategies before scaling up and/or spreading more broadly.

In both frameworks, the third phase, “going full-scale”^1^ or “national roll-out effort”,^23^ describes an effort that includes many organizations. Both frameworks present this final phase as a single phase, but both theory and evidence suggest that at the end of this phase some sites may be harder to engage.^24–26^ These late and non-adopters are typically not the focus of work published in this area;^27^ however, as efforts to expand the reach of scale-up and spread efforts grow, these sites will often be the final hurdle with which spread initiators will need to contend. We will be using the term “hard-to-engage” as a generic term to describe the group of organizations that scale-up and spread efforts have struggled to reach. This may include low performers, but these two groups are not synonymous as much as highly overlapping. As consolidations and mergers result in healthcare systems with more sites and expansive geographic boundaries, lessons about scale-up and spread from the Veterans Health Administration (VA), the largest nationwide system, become especially relevant.

The objective of this study is to describe the process of large magnitude scale-up and spread, including strategies available to scale-up and spread clinical and administrative practices across large healthcare systems, with a focus on hard-to-engage sites. Since there is a lack of information about how to tailor approaches to these hard-to-engage sites, our study explored the commonalities or characteristics of hard-to-engage sites to ascertain how these characteristics may aid or impede the spread process and explored the various strategies that have been used with hard-to-engage sites.

## METHODS

The original report commissioned by the Department of Veterans Affairs,^28^ of which this work is one part, was intended to inform ongoing national spread efforts grappling with their approach to reaching hard-to-engage sites. Given the likely paucity of literature directly addressing strategies available to scale-up and spread clinical and administrative practices—both generally and with a focus on hard-to-engage sites—we planned our approach to use a systematic search to identify literature and then augmented this with semistructured interviews to collect relevant data. We then synthesized these two data sources using content analysis.^29^

### Literature Searches

We searched multiple databases using key terms related to scaling or spread of health interventions, improving low-performing organizations, and learning health system(s). Our searches included the following databases: PubMed (inception to January 3, 2018), WorldCat (inception to January 10, 2018), Web of Science (inception to January 3, 2018), Business Source Complete (inception to November 21, 2017), SCOPUS (inception to January 10, 2018), and ROCS. We also searched for similar articles for 5 key publications.^12, 30–33^ See full search strategy in the ESM—Appendix A. In addition, we accessed the VA Assessment and Research Reporting Tool through 2017, a national database that supports administrative processes and reporting capabilities for a variety of VA research data, to find any publications affiliated with VA research projects. These publications were included in all screening and abstraction procedures.

### Literature Selection and Data Abstraction

Three reviewers independently screened the titles of retrieved citations. For citations deemed relevant by at least one person, abstracts were screened independently in duplicate. Full-text review and data abstraction was conducted independently in duplicate, with all disagreements resolved through discussion. Studies were excluded at either the abstract or the full-text level if they were not about a healthcare delivery system, about low-income country settings, about learning healthcare systems but not spread, only discussed spread conceptually, or included fewer than 10 sites in the spread effort, since these would be describing the first two phases of scale-up and spread efforts, rather than a “national roll-out effort.”^23^

For each included publication, we abstracted data on the following: the rationale for starting the spread effort, focus/topic area of the practice or initiative, where spread occurred, if and how the publication described working with hard-to-engage sites, and magnitude of spread.

### Key Informant Interview Sampling and Data Collection

In order to conduct interviews concurrently with the literature review process, we used a database with detailed project activity descriptions of all projects funded by the VA Quality Enhancement Research Initiative (QUERI) programs from fiscal years 2008 to 2012 to identify a purposeful sample of interviewees.^10^ We identified 35 projects, from a total of 82, that described scale-up or spread activities. Of these, 11 projects described conducting national, multiregional, or multisite spread as part of the scope of the project; 14 projects described evaluations of national policy or program spread efforts; and 10 projects described analyses or work with low-performing sites. We identified the most relevant projects based on their size and any specific references to spread activities being analyzed or implemented. We selected the 2 national spread projects, 2 additional multisite/multiregion projects, 3 evaluation projects, and one analysis of low-performing sites. Key informants from all 8 of the projects were contacted via email and agreed to be interviewed by phone, and they shared their perspectives on and experiences with strategies to scale-up and spread clinical and administrative practices across healthcare systems, with a focus on “hard-to-reach” sites.

The interview guide (ESM—Appendix B) was developed to focus on areas that were described in less detail in the literature, in order to have complimentary data to that from the literature. The semistructured interviews were audio-recorded and transcribed verbatim, ranging in duration from 26 to 53 min.

### Data Synthesis and Analysis

We first analyzed the literature and interview data separately, and then synthesized across these data sources using content analysis.^29, 34^ We built our analytic frame from existing frameworks and literature on scale-up and spread and identified extensions as these processes relate to hard-to-engage sites, drawing primarily on matrix analysis approach^35, 36^ which permits detailed cross-case analysis ^35, 36^. Based on our interview guide, we developed a template to rapidly organize data by interview questions.^37^ Each interview was coded by 3 members of the team, and consistency of interpretation was regularly maintained through team discussion.

## RESULTS

**Figure 1:**
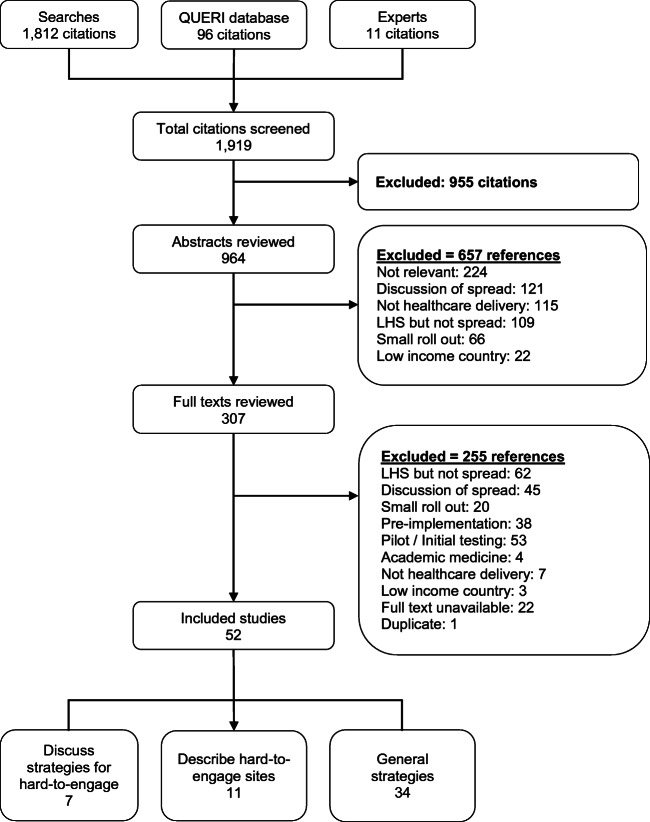
Literature flowchart.

We identified 1919 potentially relevant citations, of which 52 publications were included in this review (Fig. 1). The included publications discussed specific spread strategies for hard-to-engage sites (*n* = 7), described hard-to-engage sites but did not discuss specific strategies (*n* = 11), and discussed spread strategies more generally (*n* = 34). Table 1 includes more details about the included publications.

**Table 1:** Roll-Out Characteristics of Included Publications

Author, year	Focus area/topic	Size of roll-out Setting
Discussed hard-to-engage strategies (*n* = 7)		
Cheyne, 2013^45^	Keeping Childbirth Natural and Dynamic (KCND), a maternity care program that aimed to support normal birth by implementing multiprofessional care pathways and making midwife-led care for healthy pregnant women the national norm	NHS, Scotland Scotland
Gardner, 2010^46^	The Audit and Best Practice for Chronic Disease (ABCD) project	12 indigenous primary healthcare services in the Northern Territory of Western Australia
Lorig, 2004^39^	The six-week peer-led Chronic Disease Self-Management Program	10 of 12 regions within Kaiser Permanente USA
Lustig, 2016^26^	Measure Up/Pressure Down hypertension control campaign	Summit Medical Group (SMG) and Cornerstone Health Care (CHC) USA
Patel, 2016^25^	HPV vaccination program	23 provinces Argentina
Della Penna, 2009^47^	Implementation of a consultative model of interdisciplinary, inpatient-based palliative care (IPT)	7 of 8 regions, Kaiser Permanente USA
Robert, 2011^27^	The “Productive Ward,” a national quality improvement program	10 strategic health authorities (SHA), NHS UK
Described hard-to-engage sites (*n* = 11)		
Clarke, 2014^48^	The National Dementia Strategy for England	40 NHS sites UK
Damschroder, 2013^42^	MOVE! weight management program	55 medical centers and 872 community-based outpatient clinics VA
Hung, 2017^49^	LEAN redesign in clinic	All primary care in Sutter Health (13 sites) USA
Marshall, 2014^50^	Chronic obstructive pulmonary disease (COPD) quality improvement program	189 general practices in 4 Northeast London boroughs UK
McMullen, 2015^51^	HIV testing	40 of 45 practices in a London borough the UK
Nolan, 2005^3^	Advanced Clinic Access (ACA) initiative to reduce waiting times for patients	National VA
Noyes, 2014^52^	Nurse-led implementation, optimization, and evaluation of a complex children’s continuing-care policy	12 sites within the NHS UK
Rogers, 2014^53^	The Society of Hospital Medicine’s Glycemic Control Mentored Implementation (GCMI)	114 sites within Society of Hospital Medicine’s network USA
Parv, 2016^54^	A national e-prescription service	National Estonia
Pearce, 2014^55^	Personally controlled electronic health record (PCEHR)	74 practices across metro Melbourne Australia
van Schendel, 2017^56^	Non-invasive prenatal testing (NIPT) for aneuploidy in prenatal healthcare	National (8 medical centers) the Netherlands
Discussed spread strategies more generally (*n* = 34)		
Azar, 2015^57^	Indiana University Center for Healthcare Innovation and Implementation Science (IU-CHIIS)	Indiana Clinical and Translational Sciences Institute, Regenstrief Institute, Inc., Indiana University School of Medicine, and their clinical healthcare partners USA
Best, 2016^58^	British Columbia Ministry of Health’s Clinical Care Management (CCM) initiative, with particular focus on sepsis; surgical checklist and surgical site infection; and venous thromboembolism (VTE)	British Columbia National
Blue-Howells, 2013^59^	Veterans Justice Programs (VJP) to address the needs of justice-involved veterans by offering services to veterans at multiple points in their involvement in the criminal justice system	National VA
Boustani, 2012^60^	Indianapolis Discovery Network for Dementia (IDND)	5 healthcare systems in Indiana, including Regenstrief Institute, Inc., and Indiana University School of Medicine USA
Box, 2009^61^	Implementation of EMR for cardiac catheterization procedures called the Cardiovascular Assessment, Reporting and Tracking (CART) system	77 hospitals, national VA
Cyr, 2009^62^	Intervention to reduce door-to-balloon (D2B) time for myocardial infarction	12 community hospitals within University of Massachusetts Memorial Health Care’s service area USA
Clark, 2014^63^	State-wide clozapine management system	Adelaide metropolitan area South Australia
Duckers, 2014^64^	Quality improvement collaboratives (QIC) involvement to predict dissemination of projects within hospitals	24 hospitals the Netherlands
Elson, 2013^65^	Athena Breast Health Network	5 University of California health systems and cancer centers USA
Goetz, 2008^66^	A system-wide intervention to improve HIV testing in the Veterans Health Administration	18 sites within southern Nevada, California VA
Grayson, 2011^67^	Australian National Hand Hygiene Initiative (NHHI); infection control initiatives	521 hospitals Australia
Harris, 2016^68^	Pediatric Rheumatology Care and Outcomes Improvement Network	17 sites USA and Canada
Hendrich, 2007^69^	Ascension Health’s “Healthcare That Works, Healthcare That is Safe, and Healthcare That Leaves No One Behind” with goal of zero preventable injuries or deaths	Ascension Health hospitals (65 sites) USA
Johnson, 2017^70^	Inflammatory Bowel Disease (IBD) Qorus learning health system	20 adult IBD care USA
Kellogg, 2017^71^	Tested a new method of intra-organizational process development and spread of quality improvement innovations	10 sites within North Shore Physicians Group USA
Kwon, 2012^72^	Washington State’s Surgical Care and Outcomes Assessment Program (SCOAP)	60 of 65 hospitals in State of Washington USA
Lannon, 2013^73^	Pediatric Collaborative Improvement Networks to improve pediatric subspecialty care	Multi-institution USA
Lennon, 2017^74^	Delivering Assisted Living Lifestyles at Scale (dallas), a national digital health program	NHS UK
Liu, 2016^38^	Quality of sepsis care	Kaiser Permanente Northern California (21 hospitals) USA
Mills, 2003^75^	Quality Interagency Coordination Task Force (QuIC) initiative to reduce medical errors	22 hospitals VA
Ovseiko, 2014^76^	Health Innovation and Education Clusters (HIECS)	NHS UK
Psek, 2015^77^	Operationalizing the learning healthcare system (LHCS) in an integrated delivery system	Geisinger Health System (8 hospitals) USA
Ramsey, 2017^78^	ImproveCareNow Network to facilitate personalized medicine for children and adolescents with inflammatory bowel disease (IBD)	92 care centers USA, England, Qatar
Resnick, 2007^79^ Resnick, 2009^80^	Supported employment for veterans	21 sites across the VA VA 166 VA medical centers VA
Rocker, 2017^81^	INSPIRED COPD outreach program	19 teams in 10 provinces Canada
Rubenstein, 2010^82^	Implementation of Translating Initiatives in Depression into Effective Solution (TIDES) aimed to translate research-based collaborative care for depression	Medium-sized primary care practices within the VA VA
Curran, 2011^83^	Implementation of collaborative care for depression in HIV clinics (HIV Translating Initiatives for Depression into Effective Solutions, HITIDES)	3 sites VA
Luck, 2009^84^	Implementation of Translating Initiatives in Depression into Effective Solution (TIDES) aimed to translate research-based collaborative care for depression	National VA
Sherman, 2007^85^	Implementation of Translating Initiatives in Depression into Effective Solution (TIDES) aimed to translate research-based collaborative care for depression	National VA
Smith, 2008^86^	Development of a national dissemination plan for collaborative care for depression	National VA
Schmittdiel, 2017^87^	The Delivery Science Rapid Analysis Program (RAP)	Kaiser Permanente in northern California USA
Septimus, 2016^88^	Implementation of universal decolonization to reduce healthcare-associated central line-associated bloodstream infections (CLABSI)	136 ICUs in 95 hospitals affiliated with Hospital Corporation of America USA
Yano, 2015^89^	The Collaborative Research to Advance Transformation and Excellence (CREATE) Initiative for comprehensive care for women veterans	National VA

### Breaking Down the National Scale-Up or Spread Process

**Figure 2:**
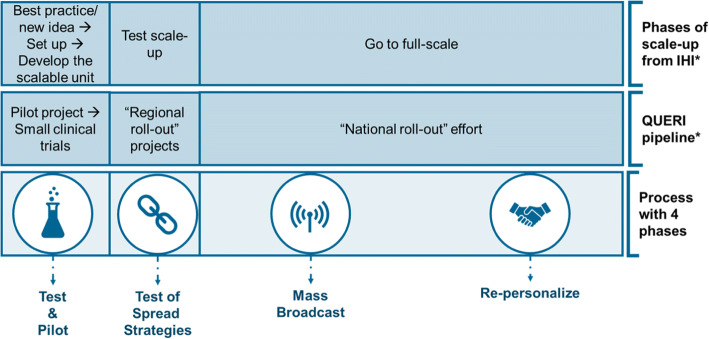
Breaking down the national scale-up or spread process. * IHI phases of scale-up^1^ and QUERI pipeline.^21^

The literature and interview data supported the descriptions of scale-up and spread proposed by the QUERI pipeline^21^ and IHI phases of scale-up^1^ in the first two phases, but our data split the final phase of “going full-scale”^1^ or “national roll-out effort”^23^ into two parts with distinct strategies which we describe as “mass broadcast” and “re-personalization” (see Fig. 2).

#### Mass Broadcast

The first part of the full-scale spread, which we are calling the “mass broadcast” phase, uses strategies intended to reach maximal audience. This first part seems to align with descriptions from the frameworks.^1, 23^

In publications and interviews alike, this phase was nearly always described as beginning with strong top-down support:

“…having a strong partnership with [national leaders] was a critical factor in making this happen and getting the facilities involved because they knew that we had the backing of the National Program Office.”

This top-down support could take the form of summits with all top-level leadership, for example: “… senior regional leadership identified reducing sepsis mortality as a key performance improvement goal… The effort was launched… at a Sepsis Summit.”^38^ Other more formal arrangements like an official mandate or policy change were also used, with mandates present cited in nearly every interview. This top-down support was typically effective during the “mass broadcast” phase of national spread efforts.

#### Re-personalization

The second part of full-scale spread, which we are calling the “re-personalization” phase, is focused on hard-to-engage sites that did not engage at the “mass broadcast” stage. The strategies recommended for hard-to-engage sites reflect a return to a more personalized approach, which uses more direct connection akin to what is typical in the first two phases of scale-up and spread. Early in the spread process, when experimenting with and testing strategies, spread initiators usually engage sites to collect data, refine approaches, and learn from experiences.

### Considerations and Strategies for Working with Hard-to-Engage Sites

**Figure 3:**
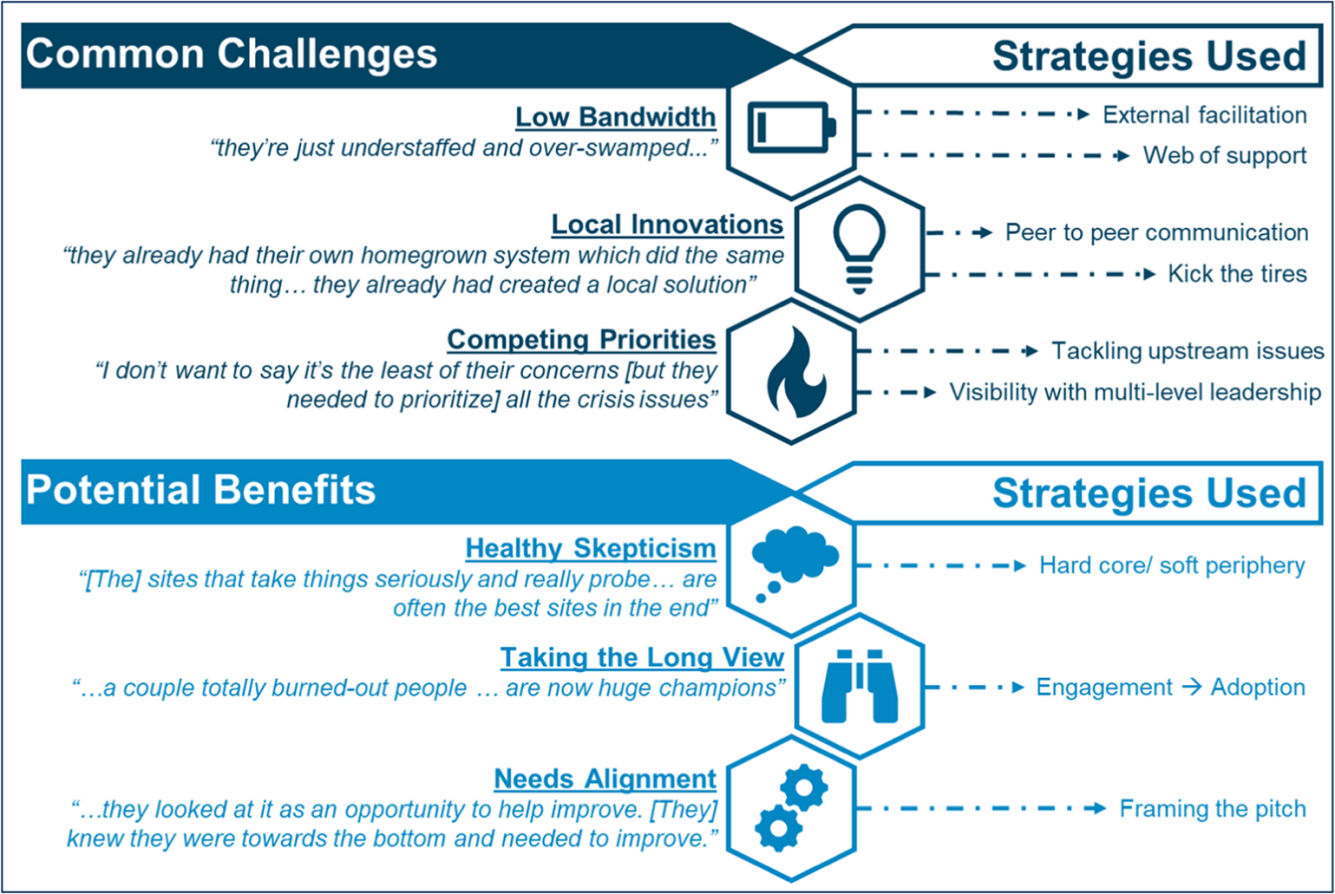
Considerations and strategies for working with hard-to-engage sites.

We drew from interviews and from the 18 publications we identified as either providing descriptions of hard-to-engage sites only (*n* = 11) or additionally providing descriptions of strategies used with these hard-to-engage sites (*n* = 7). Interviewees and publications alike supported the highly context-specific nature of challenges faced by hard-to-engage sites, whose “problems vary tremendously” with a “myriad of individual reasons,” according to interviewees. The phrase “N-of-1” was used repeatedly by interviewees to describe experiences working with hard-to-engage sites. Since hard-to-engage sites are highly variable in their needs, interviewees recommended “a flexible, tailored approach to one [site] at a time.” Drawing from both interviews and literature, we describe useful strategies to address these common challenges and maximize potential benefits (Fig. 3).

#### Common Challenges for Spreading to Hard-to-Engage Sites and Strategies for Addressing Them

Certain challenges arise that spread initiators or sites themselves may face when working with hard-to-engage sites (see Table 2). Spread initiators described a variety of approaches tailored to hard-to-engage sites that faced common challenges (see Table 3).

**Table 2:** Benefits of and Challenges of Spreading to Hard-to-Engage Sites

Characteristic	Interviews	Publications
Common challenges for spreading to hard-to-engage sites		
Limited bandwidth or resources	• They’re just understaffed and over-swamped... they recognize that it’s a great program that they want to do with us, so we’re trying to help them navigate through all their other stuff. • [Participants say] “I can't take one more thing.” … it gets so difficult to do [the initiative].”	• Locally, sites often encountered resource shortages … The failure … to complete the program was often due to lack of administrative support, time constraints, departure of key team members from their institutions, and difficulty engaging a multidisciplinary team.^53^ • Lack of resources was frequently mentioned. Resource constraints sometimes necessitated a team that lacked one or more professional disciplines (i.e., physician, nurse, chaplain, social worker), rotated different providers through the IPC team, or operated less than full time. As a senior leader noted, “It has been hard for the regions and the medical centers to find the funds.”^47^ • Another senior leader commented, “[Resources] weren’t enough to cover the model, so we had to scramble and shift things around to be as true as we could to the model.”^47^
Local innovations	• It doesn’t mean they were low quality sites, though, but that they’re just last sites to adopt. In some ways they were often high-quality, forward-thinking sites that had already tried to solve the solution, but they were laggards in terms of adopting [the initiative]…they already had their own homegrown system which did the same thing roughly… they already had created a local solution.	• There was no expressed need for the program.^42^ • Sites with pre-existing [programs] tended to move more slowly to adopt.^47^
Competing priorities	• [Sites] “in extremis” that are falling apart don’t really want to do anything… they’re concerned with getting through the day,” • [Some of the hard-to-engage sites] that are otherwise big academic places… they’re focused on something for themselves…[the initiative is] I don’t want to say it’s the least of their concerns because it’s a very important problem, but… with all the crisis issues…they don’t seem to be as engaged.	• Low implementation facilities were struggling to respond to other higher priority initiatives,… It just depends on where you are on the totem pole.^42^ • Sites often encountered [challenges] because of competing organizational initiatives and a lack of prioritization…at the level of the executive suite.^53^
Benefit of working with hard-to-engage sites		
Healthy skepticism	• They are activated and I think in it to win it for their patients…so yeah, not constipators—if anything, they’d be active resistors if they could be, and it’s a different kind of thing. • It can be a way to engage a site by letting them in on what you find and getting their perspectives on what might help…What appear to be difficult sites are often sites that take things seriously and really probe.	
Taking the long view	• I dealt with a couple totally burned-out people who are now huge champions	• The region that decided to postpone implementation benefitted from the experience of the other regions in working out issues… [they] joined the monthly conference calls and asked many clarifying questions regarding the issues being discussed… [this region] waited and then built a strong base of support for the program… chose to take time to build organizational readiness… when they implemented the program…they were successful.^39^ • The advantage of later joiners… was that they could draw on and gain support from the experience of early enrollees.^46^
Alignment with needs	• There are some who want to hide their low-performance status and there are others who want to really get better and take advantage of a learning community and work on it and improve. • They close themselves off from the outside world because…they can’t take on…you can get to the right people that you potentially could help them, say with somebody there locally recognizes, hey, wait a minute, this might be something that could actually help us…[low-performers] have been one of the easier ones because they looked at it as an opportunity to help improve. This is a group that knew they were towards the bottom and needed to improve.

**Table 3:** Strategies for Working with Hard-to-Engage Sites

Strategy	Interviews	Publications
Strategies for addressing common challenges in spreading to hard-to-engage sites		
External facilitation	• We did have these facilitation phone calls where someone led and checked in with the status of making sure that the clinical reminder was on, making sure that they were trying to do nurse standing orders, where they were with that, what assistance they needed with it; so kind of troubleshooting to make sure that things were moving forward with getting patients vaccinated. • Having regular calls was critical to them just kind of keeping one foot in front of the other…they were like “Thank you for giving me the space. Even though it was squeezed in the margins, you were willing to spend a half-hour call at the end of the day.”	• Iterative quality improvement processes were supported by… the national team.^47^
“Web of support”	• You kind of have to create a web of support around trying to work these things through. So it’s never good to have a single person be your point person in many of these places. • What we found and what would happen for that clinical champion is that other team members were able to step in and actually wanted to communicate directly with that with us. So we didn’t have to just tunnel through the site leads. The other team members were very comfortable with that because we did go on site for like the big kickoff. So I felt like they got to know us and the other team members were comfortable talking with us as much as the team leaders.	
Peer-to-peer communication	• It truly is an interactive collaborative. And we do get representation from all the six sites into the call so we’re very pleased with that. The best part of it is really when teams talk to each other. • Although the VISN[s] started out somewhat different in what they were going to do, over time they came to very similar models…they kind of stole from each other, which was great. • Let me emphasize the words ‘peer-to-peer,’ they have to be on the same exact level and view those people as peers.	• [The initiative] benefited from champions in each respective practice and specialty to ensure that buy-in was achieved in all facets of the organization.^26^
“Kick the tires”	• As one was refusing to even participate in the project but I finally asked her, “Why would you not want to implement evidence?... [try it] if you don’t like it, you can walk away.” And she turned out to be our biggest champion. • It’s not perfect, but let’s walk you through it. Here’s how to use it. Hopefully it’s pretty straightforward. Give me any feedback you have… so what we’re asking you to do is take it, use it, either on test cases, just practice with it, or start to deploy it in real reporting. But kick the tires…over many years [the team had] a mechanism of feedback from the field, from the users… we had a workgroup of peers for the community… and we rotated them, by the way, every couple years so that lots of people could get experience across the system in this… these small iterative version of the changes that would then get implemented nationally.	
Tackling upstream issues	• Some units didn’t know how to download … a mailing list with labels … so we had to help them work through how to be able to do those types of activities…some places had some issues … getting their [IT] to work with them. • [Sites] tend to not have a lot of organizational slack…[they may] not have as much of a quality improvement or system redesign infrastructure. • We’re trying to help them navigate through all their other stuff. And they are making an effort… So among all their other activities and other requirements, we’re trying to help them participate and do the work. • We’ll collect the data, we’ll analyze it, we’ll present it, we’ll go fight the battles for you.	
Increasing visibility with multiple levels of leadership	• We’ve also given a lot of materials to show for our clinical champions to share with their leadership to show that look at the good work we’re doing…[and] they’ve gotten great direct feedback from the administration that they were very supportive and they’re very congratulatory of the work they’re doing. • We also give them a voice with leadership above…so I think what we’re kind of referring to as the multilevel stakeholder engagement piece becomes really important, and then having a communications plan from the local folks on up to the [regional] level and up to the medical center level, and in some cases all the way up to [national] levels, becomes really important…Part of a very engaged executive steering committee, and so we would be feeding results back to them on quite a regular basis.	• Having the involvement of multiple levels of leadership creates a snowball effect throughout an organization and is a significant contributor to Measure Up/Pressure Down’s success.^26^
Strategies for maximizing potential benefits of working with hard-to-engage sites		
Hard core and a soft periphery	• We called it a multipronged intervention, but everyone didn’t do the same thing. And we had all these strategies: clinical reminder, nurse standing orders, education, doing the mailings, all of that…they did it if they wanted to do it. • A small bit of customization, but all the core [pieces were standardized]. • It’s not one-size-fits-all. They have room to adapt. • This whole sort of Evidence-Based Quality Improvement approach is to be responsive to the time and the situation… it was really designed to get a lot of input both at the [regional] level and at the site level in how to adapt or tailor. And it sort of started with an agreement that the ultimate models at the sites would reflect the key elements of the literature in areas that the literature addressed, but that outside of those kind of pillars, the project model would be shaped by the sites themselves.	• [The] hard core provides a standardized method… the soft periphery…adapted by organizations in different ways to maximize fit in the local context and to build acceptability among staff.^46^
Maintaining engagement	• If the sites continue to be engaged, I have found that those sites are often the best sites in the end.	• By directly addressing concerns…[they] built up a community of people who could further advocate for the use of the vaccine.^25^ • The region that did not initially start the [program] with other regions… [had a] regional representative joined the study’s monthly conference calls.^39^
Framing the pitch	• The top-down or other mechanisms of engagement… always come across as punishment…[sites] have the best insight into their own things and what they might want to be able to do, [so engaging in a discussion has more positive results]. • I get the sense often that people feel blamed for their problems rather than being made to feel part of the solution, and I think where I feel I have more success is when I’m working with people who take pride in being part of the solution. • The education focus included meetings that I would say resulted in [relationships] ...Education included brining people in to talk….just to see what happen, basically raising awareness of the issue…once people will let themselves realize that there’s a problem and the scope of the problem, suddenly they become [willing to engage]. • Somebody there locally recognizes, hey, wait a minute, this might be something that could actually help us. So it’s a little bit of social marketing. And whether you can do that, again, with numbers, definitely trying to communicate to the administration what your intentions are nobody wants to be pointed out again that they’re not doing well. So then you actually might be able there to help.	

Limited bandwidth or resources, such as turnover and lack of funding, burnout, or implementation as an added duty without additional compensation,^39^ were common in hard-to-engage sites. No system or model of spread seemed to be immune, as “lack of resources” was frequently mentioned as a factor impeding spread.^49^ One strategy spread initiators used for hard-to-engage sites with limited resources was external facilitation,^40, 41^ which provides additional supports to those sites with low bandwidth, or who may need extra support for other reasons. Working with multiple local people reduces the burden on any individual and strengthens overall linkages to that site for a spread initiative. This strategy provides a “web of support,” as one interviewee called it.

Local innovations or homegrown solutions to the same problem can present competition that impedes spread, since “there was no expressed need for the program.”^42^ Two strategies to mitigate this challenge are (1) peer-to-peer communication, where individuals share information and receive support from fellow spread initiative participants, particularly from individuals of the same “rank” or “level”, and (2) to allow local sites to “kick the tires” of the innovation, which gives sites a chance to test the innovation and provide feedback prior to implementation (i.e., “trialability”).^24^

Potential spread sites were often very busy addressing local priorities that may not overlap with the aims of a particular spread initiative. Although competing priorities can impede scale-up and spread, tackling upstream issues, such as pre-existing information technology infrastructure gaps, and increasing visibility with multiple levels of leadership can help protect the initiative and demonstrate success for those sites involved.

#### Potential Benefits of Working with Hard-to-Engage Sites and Strategies to Maximize Them

Spread initiators identified several ways that they perceived hard-to-engage sites would view participating in spread initiative as beneficial, and, while slower to start, these sites reaped unique benefits for themselves (see Table 2). In working with hard-to-engage sites, spread initiators described using a few strategies that maximized engagement and, in turn, potential benefits (see Table 3).

Interviewees described situations where “healthy skepticism” led to collaboration and, in some cases, improvement of the practice or initiative being spread. Taking advantage of a “hard core and a soft periphery”^43^ model of intervention, where the core model is adaptable to a local context, may help realize local compatibility and fit needs that may differ from sites where the intervention was originally tested.

Some spread initiators chose to “take the long view” with the scale-up and spread process. They noted that once some hard-to-engage sites are engaged, their hard-won adoption could lead to more sustainable successes in the long-term, in contrast with early adoption which could lead to superficial engagement and, consequently, abandonment. For these long-term wins, spread initiators maintained engagement and gave opportunities for slower adopters to build commitment and find avenues to adoption within their local contexts.

There is added incentive for sites to participate in a spread initiative when goals of spread efforts align with the needs of hard-to-engage sites. In framing the pitch, establishing rapport with hard-to-engage sites early in the process by conducting in-person initial visits could help with spread initiative. Interviewees consistently described focusing on “being seen” as helpful, rather than punitive or authoritarian action.

## DISCUSSION

Using content analysis of literature and key informant interviews, we described four phases of scale-up and spread, the first two aligning with descriptions presented in QUERI and IHI frameworks. We suggest that rather than one more phase of a “national roll-out effort,”^23^ there is a third phase, mass broadcast, in which strategies are used to reach maximal audience, and a fourth phase, re-personalization, marked by a return to using strategies more often employed in the early phases of the spread process. While descriptions of hard-to-engage sites often portrayed challenges, a number of beneficial characteristics were also depicted. Hard-to-engage sites can be highly variable in terms of the challenges or barriers they face. Since hard-to-engage sites are heterogeneous in their needs, interviewees recommended “a flexible, tailored approach to one [site] at a time.”

While many frameworks and models exist that outline scale-up and spread in a general way, scant published evidence has been identified that provides discussion of strategies for reaching sites that are hard-to-engage. Those publications that did mention hard-to-engage sites spent a few sentences, at most, discussing the topic. The sparse data identified from the literature aligned with key informant accounts, which allowed us to differentiate this last group in a fourth phase. This finding expands on prior discussions of scale-up and spread and is hypothesis generating, and while some promising new work is underway,^44^ more studies focused in this area are needed. Additional exploratory studies could determine if there is consistent representation of these concepts in scale-up and spread efforts, and better documentation of the later stages of spread efforts, including specific strategies and/or adaptations used to engage hard-to-engage sites, is needed.

Because terminology related to scale and spread is evolving, there are no reliable, standardized terms for systematically searching for literature related to this topic, so relevant literature might have been missed. In addition, studies that have conducted large magnitude scale initiatives do not always describe their experiences with or strategies for engaging hard-to-engage sites. We also do not have information about the contexts or success of unpublished spread efforts, of which there are likely many, given that spread and scale-up happens regularly in nonresearch settings. While interviews give a depth of information, we were not able to gather that detailed data for all initiatives identified in the literature synthesis portion of the study. While other initiatives, particularly those outside the VA, may have different experiences to report, the data we did have aligned well, independent of setting. In addition, we limited our scope to scale-up and spread in healthcare settings, given that the nature of initiatives in healthcare settings tend to be complex, which mirrors the complexity of the services provided in healthcare settings compared to other industries. Additionally, healthcare organizations tend to be very large, have many layers of hierarchy and authority, and be subject to unique regulation and policy pressures and other factors that make scale-up and spread efforts in this field unique. However, spread in other nonhealthcare settings could potentially inform healthcare spread, and our current scope would not have included these potentially relevant experiences.

If implementation science is to expand its impact in routine care settings, more testing of scale-up and spread strategies, as well as documentation of adaptations or tailoring, is needed. Hard-to-engage audiences are most in need of engagement when spreading innovations intended to standardize practice or improve quality of care, but they are understudied. Ameliorating variations in care delivery will require a better understanding of how to work with hard-to-engage groups. For the myriad of individual factors these sites face, bundles of engagement strategies that are more personalized and intensive seem to help spread initiators reach these groups, but determining which strategies work well in different situations will require additional empirical work.

## Electronic Supplementary Material

ESM 1 (DOCX 27 kb)
