# Supplementary material for: Scaling Beyond Early Adopters: a Content Analysis of Literature and Key Informant Perspectives
Source: J Gen Intern Med. 2020 Oct 27;36(2):383–95. doi: 10.1007/s11606-020-06142-0 (PMC7878615; doi:10.1007/s11606-020-06142-0)
Supplement: Supplementary file 1 — (DOCX 27 kb) [file 11606_2020_6142_MOESM1_ESM.docx]

AppendiX A. Search Strategy

**TOPIC 1 – SCALING/SPREAD OF HEALTH INTERVENTIONS**

**TOPIC 2 – IMPROVING LOW-PERFORMING ORGANIZATIONS**

**TOPIC 3 – LEARNING HEALTHCARE SYSTEMS**

**SEARCH TOPIC 1 – SCALING/SPREAD:**

**DATABASE SEARCHED & TIME PERIOD COVERED:**

PubMed – From inception to 1/4/2018

**LANGUAGE:**

English

**SEARCH STRATEGY:**

scale-out[tiab] OR scaling-out[tiab] OR scaling[ti] OR scaling-up[tiab] OR scale-up[ti] OR spread*[ti] OR spread*[ot] OR scale-out[ot] OR scaling-out[ot] OR scaling[ot] OR scaling-up[ot] OR scale-up[ot] OR large-scale OR "large scale" OR national[ti] OR system-wide OR "system wide" OR multi-institutional system* OR "Multi-Institutional Systems"[Mesh]

AND

"organizational culture"[ti] OR "organisational culture"[ti] OR organizational chang*[ti] OR organisational chang*[ti] OR organizational innovat* OR "diffusion of innovation"

AND

interven*[tiab] OR interven*[ot] OR initiative*[tiab] OR initiative*[ot] OR implement* OR practice[tiab]

--------------------------------------------------------------------------------------------------------------------------

**DATABASE SEARCHED & TIME PERIOD COVERED:**

PubMed – From inception to 1/4/2018

**LANGUAGE:**

English

**SEARCH STRATEGY:**

SIMILAR ARTICLE SEARCHES –

Aarons, Gregory A.

"Scaling-out" evidence-based interventions to new populations or new health care

delivery systems.

Implement Sci. 2017 Sep 6;12(1):111.

Yano, Elizabeth M

Implementation and spread of interventions into the multilevel context of routine

practice and policy: implications for the cancer care continuum.

J Natl Cancer Inst Monogr. 2012 May;2012(44):86-99

----------------------------------------------------------------------------------------

**DATABASE SEARCHED & TIME PERIOD COVERED:**

WorldCat – From inception to 1/3/2018

**LANGUAGE:**

English

**SEARCH STRATEGY:**

ti: scale-out OR ti: scaling-out OR ti: scaling OR ti: scaling-up OR ti: scale-up OR ti: spread* OR ti: large-scale OR ti: large w scale OR ti: system-wide OR ti: system w wide OR ti: multi-institutional w system OR ti: multi-institutional w systems)) or (su: scale-out OR su: scaling-out OR su: scaling OR su: scaling-up OR su: scale-up OR su: spread* OR su: large-scale OR su: large w scale OR su: system-wide OR su: system w wide OR su: multi-institutional w system OR su: multi-institutional w systems)) not mt: juv) not mt: fic and (dt= "bks" or dt= "ser" or dt= "url")

AND

ti: medical OR ti: health* OR ti: hospital OR ti: hospitals

AND

ti: chang* OR ti: innovat* OR ti: implement* OR ti: initiative* OR ti: interven* OR ti: cultur* or su: chang* OR su: innovat* OR su: implement* OR su: initiative* OR su: interven* OR su: cultur*

AND

ti: quality OR ti: improv* or su: quality OR su: improv*

-------------------------------------------------------------------------------------------------------------------------------

**DATABASE SEARCHED & TIME PERIOD COVERED:**

Web of Science – From inception to 1/3/2018

**LANGUAGE:**

English

**SEARCH STRATEGY:**

ti=(scale-out OR scaling-out OR scaling OR scaling-up OR scale-up OR spread* OR large-scale OR large near scale OR system-wide OR system near wide OR multi-institutional near system OR multi-institutional near systems)

AND

ts=(medical OR health* OR hospital OR hospitals)

AND

ts=(chang* OR innovat* OR implement* OR initiative* OR interven* OR culture*)

AND

ti=(quality OR improv*)

Refined by: [excluding] WEB OF SCIENCE CATEGORIES: ( FOOD SCIENCE TECHNOLOGY OR GREEN SUSTAINABLE SCIENCE TECHNOLOGY OR URBAN STUDIES OR ENVIRONMENTAL SCIENCES OR VETERINARY SCIENCES OR BIOCHEMICAL RESEARCH METHODS OR BIOCHEMISTRY MOLECULAR BIOLOGY OR BIOLOGY OR EDUCATION SCIENTIFIC DISCIPLINES OR BIOPHYSICS OR ENERGY FUELS OR BUSINESS OR ENVIRONMENTAL STUDIES OR BUSINESS FINANCE OR METEOROLOGY ATMOSPHERIC SCIENCES OR CELL BIOLOGY OR MULTIDISCIPLINARY SCIENCES OR CHEMISTRY MULTIDISCIPLINARY OR COMPUTER SCIENCE ARTIFICIAL INTELLIGENCE OR AGRICULTURE DAIRY ANIMAL SCIENCE OR COMPUTER SCIENCE HARDWARE ARCHITECTURE OR AGRICULTURE MULTIDISCIPLINARY OR COMPUTER SCIENCE INFORMATION SYSTEMS OR AUDIOLOGY SPEECH LANGUAGE PATHOLOGY OR COMPUTER SCIENCE SOFTWARE ENGINEERING OR SPORT SCIENCES OR BIOTECHNOLOGY APPLIED MICROBIOLOGY OR CONSTRUCTION BUILDING TECHNOLOGY OR CHEMISTRY PHYSICAL OR CRYSTALLOGRAPHY OR COMPUTER SCIENCE INTERDISCIPLINARY APPLICATIONS OR EDUCATION SPECIAL OR COMPUTER SCIENCE THEORY METHODS OR ELECTROCHEMISTRY OR DEMOGRAPHY OR ENGINEERING CIVIL OR WATER RESOURCES OR ENGINEERING ELECTRICAL ELECTRONIC OR EDUCATION EDUCATIONAL RESEARCH OR ENGINEERING MANUFACTURING OR ECOLOGY OR ENGINEERING MECHANICAL OR GEOGRAPHY OR ETHICS OR EVOLUTIONARY BIOLOGY OR MARINE FRESHWATER BIOLOGY OR FORESTRY OR MATHEMATICS INTERDISCIPLINARY APPLICATIONS OR GENETICS HEREDITY )

OR

ts=(implementation science) AND ts=(system* near chang*)

Refined by: WEB OF SCIENCE CATEGORIES: ( NEUROSCIENCES OR HEALTH CARE SCIENCES SERVICES OR IMMUNOLOGY OR HEALTH POLICY SERVICES OR MEDICINE GENERAL INTERNAL OR PSYCHOLOGY CLINICAL OR PSYCHOLOGY DEVELOPMENTAL OR PSYCHOLOGY EDUCATIONAL OR PSYCHOLOGY MULTIDISCIPLINARY OR ONCOLOGY OR PHARMACOLOGY PHARMACY OR SOCIAL SCIENCES BIOMEDICAL OR CLINICAL NEUROLOGY OR MEDICAL INFORMATICS OR SOCIAL SCIENCES INTERDISCIPLINARY OR HEMATOLOGY OR INFECTIOUS DISEASES OR SOCIAL ISSUES OR MEDICINE RESEARCH EXPERIMENTAL )

------------------------------------------------------------------------------------------------------------------------**DATABASE SEARCHED & TIME PERIOD COVERED:**

Web of Science – From inception to 1/4/2018

**LANGUAGE:**

English

**SEARCH STRATEGY:**

**“Forward” search on the following article:**

Yano, Elizabeth M

Implementation and spread of interventions into the multilevel context of routine

practice and policy: implications for the cancer care continuum.

J Natl Cancer Inst Monogr. 2012 May;2012(44):86-99

**==========================================================================**

**SEARCH TOPIC 2 – LOW-PERFORMING ORGANIZATIONS**

**DATABASE SEARCHED & TIME PERIOD COVERED:**

PubMed – From inception to 11/21/2017

**LANGUAGE:**

English

**SEARCH STRATEGY #1 (ORIGINAL VERSION)**

organization* AND perform*[ti]

AND

low OR lower OR lowest OR low-perform* OR poor* OR substandard

AND

interven* OR improv*

------------------------------------------------------------------------------------------------------------------------**DATABASE SEARCHED & TIME PERIOD COVERED:**

PubMed – From inception to 1/3/2018

**LANGUAGE:**

English

**SEARCH STRATEGY #2 (REVISED VERSION)**

low perform* OR low-perform* OR lower perform* OR lower-perform* OR lowest perform* OR lowest-perform* OR perform* poor*

AND

"organizational culture"[ti] OR "organizational culture"[mh] OR "organisational culture"[ti] OR organizational chang*[ti] OR organisational chang*[ti] OR organizational innovat* OR "diffusion of innovation"

-------------------------------------------------------------------------------------------------------------------------------**DATABASE SEARCHED & TIME PERIOD COVERED:**

Business Source Complete – From inception to 11/21/2017

**LANGUAGE:**

English

**SEARCH STRATEGY:**

SU organizational performance

AND

TI ( low OR lower OR lowest OR low-perform* OR poor* OR substandard )

AND

interven* OR improv*

Search modes - Find all search terms

**==========================================================================**

**SEARCH TOPIC 3 – LEARNING HEALTHCARE SYSTEMS**

**DATABASE SEARCHED & TIME PERIOD COVERED:**

PubMed- From inception to 1/10/2018

**LANGUAGE:**

English

**SEARCH STRATEGY:**

learning health system* OR learning healthcare system* OR "learn from every patient"

OR

("learn from every patient" OR lfep) AND ("nationwide children's hospital" OR "nationwide childrens hospital"

OR

**“SIMILAR ARTICLE” SEARCHES ON THE FOLLOWING ARTICLES:**

Grumbach, Kevin,“Transforming from centers of learning to learning health systems: the challenge for academic health centers,” JAMA. 2014 Mar 19;311(11):1109-10.

Lowes, Linda P.,“Learn From Every Patient': implementation and early results of a learning health System,” Dev Med Child Neurol. 2017 Feb;59(2):183-191.

Smoyer, William E.,“Creating Local Learning Health Systems: Think Globally, Act Locally,” JAMA. 2016 Dec 20;316(23):2481-2482.

OR

JOURNAL - "Hospitals and Health Networks" for all issues in 2017

-------------------------------------------------------------------------------------------------------------------------------

**DATABASE SEARCHED & TIME PERIOD COVERED:**

WorldCat: - From inception to 1/10/2018

**LANGUAGE:**

English

**SEARCH STRATEGY:**

kw: learning w health w system* OR kw: learning w healthcare w system* OR kw: learn w1 every w1 patient

AND

DOCUMENT TYPE= BOOKS OR SERIALS OR ARTICLES OR URL

NOT

SUBJECT= education OR MEDIA TYPE=juvenile OR MEDIA TYPE=fiction

-------------------------------------------------------------------------------------------------------------------------------

**DATABASE SEARCHED & TIME PERIOD COVERED:**

Web of Science - From inception to 1/10/2018

**LANGUAGE:**

English

**SEARCH STRATEGY #1:**

ts=("learning health system" OR "learning health systems" OR "learning healthcare system" OR "learning healthcare systems" OR "learn from every patient")

**SEARCH STRATEGY #2:**

Forward searches on Grumbach, Lowes, & Smoyer articles

-------------------------------------------------------------------------------------------------------------------------------

**DATABASE SEARCHED & TIME PERIOD COVERED:**

Scopus - From inception to 1/10/2018

**LANGUAGE:**

English

**SEARCH STRATEGY #1:**

TITLE-ABS-KEY ("learning health system" OR "learning health systems" OR "learning healthcare system" OR "learning healthcare systems" OR "learn from every patient" )

**SEARCH STRATEGY #2:**

Forward searches on Grumbach, Lowes, & Smoyer articles

-------------------------------------------------------------------------------------------------------------------------------

**DATABASE SEARCHED & TIME PERIOD COVERED:**

IEEE XPLORE - From inception to 1/10/2018

**LANGUAGE:**

English

**SEARCH STRATEGY:**

"learning health system" OR "learning health systems" OR "learning healthcare system" OR "learning healthcare systems" OR "learn from every patient"

-------------------------------------------------------------------------------------------------------------------------------

**DATABASE SEARCHED & TIME PERIOD COVERED:**

Embase - From inception to 1/10/2018

**LANGUAGE:**

English

**SEARCH STRATEGY:**

'learning health system' OR 'learning health systems' OR 'learning healthcare system' OR 'learning healthcare systems' OR 'learn from every patient’

AND

Humans

-------------------------------------------------------------------------------------------------------------------------------

**DATABASE SEARCHED & TIME PERIOD COVERED:**

ACM Digital Library - From inception to 1/10/2018

**SEARCH STRATEGY:**

"learning health system" OR "learning health systems" OR "learning healthcare system" OR "learning healthcare systems" OR "learn from every patient"

-------------------------------------------------------------------------------------------------------------------------------

**DATABASE SEARCHED & TIME PERIOD COVERED:**

CINAHL - From inception to 1/10/2018

**LANGUAGE:**

English

**SEARCH STRATEGY:**

TI ("learning health system" OR "learning health systems" OR "learning healthcare system" OR "learning healthcare systems" OR "learn from every patient" ) OR AB ( "learning health system" OR "learning health systems" OR "learning healthcare system" OR "learning healthcare systems" OR "learn from every patient" ) OR MW ( "learning health system" OR "learning health systems" OR "learning healthcare system" OR "learning healthcare systems" OR "learn from every patient")

-------------------------------------------------------------------------------------------------------------------------------

**DATABASE SEARCHED & TIME PERIOD COVERED:**

PsycINFO - From inception to 1/10/2018

**LANGUAGE:**

English

**SEARCH STRATEGY:**

TI ("learning health system" OR "learning health systems" OR "learning healthcare system" OR "learning healthcare systems" OR "learn from every patient" ) OR AB ( "learning health system" OR "learning health systems" OR "learning healthcare system" OR "learning healthcare systems" OR "learn from every patient" ) OR ( "learning health system" OR "learning health systems" OR "learning healthcare system" OR "learning healthcare systems" OR "learn from every patient")

**NOTE: RESULTS OF ORIGINAL PUBMED AND BUSINESS SOURCE COMPLETE SEARCH VERSIONS WERE REVIEWED AND ONLY SELECTED RELEVANT ITEMS WERE INCLUDED IN FINAL SET**

AppendiX B. Interview Guide Questions

**INTERVIEW GUIDE QUESTIONS – QUERI Interviewees**

*[QUESTIONS FOLLOW INTERVIEW GUIDE INTRODUCTORY SECTION]*

We understand you were the Principal Investigator for the QUERI project [PROJECT NAME]. We are particularly interested in this project because it was an example of spreading an existing project.

1. Please tell us about your experience with this project.
2. Can you describe the strategy for the spread of [INITIATIVE/PRACTICE]?
   1. Who was involved in making the decision to spread beyond the earlier sites?
   2. Who was involved in the spread effort itself?
3. What factors [national/regional/local/site specific] facilitated the spread of the project?
4. What factors [national/regional/local/site specific] impeded the spread of the project?
5. Were certain sites more difficult to engage?
   1. If so, what factors contributed to this?
   2. *Potential factors to probe: leadership, resources, lines of reporting/authority to make changes, structural factors*
      1. Was low performance a factor?
      2. Were there specific challenges?
   3. Were there specific strategies used for engaging or working with this group of sites?
6. During spread efforts, was fidelity of implementation monitored?
   1. If so, how?
   2. During spread, was fidelity to original model strong?
   3. Were modifications made to the model or strategy?
      1. If so, why?
      2. What changes to the strategy were most successful?
      3. Which were less successful?
7. From the time the idea for [INITIATIVE/PRACTICE] was first conceived, could you briefly describe the key time points in the process?

*Eg, initial idea, first piloting/demo, early spread, full/national roll-out*

Is there anything else you would like to share with us, particularly about working with hard-to-engage sites? Please feel free to draw on other experience you may have had.

Thank you for your time!
